# Exploration of genetic factors resulting in abnormal disease in cattle experimentally challenged with bovine spongiform encephalopathy

**DOI:** 10.1080/19336896.2020.1869495

**Published:** 2021-01-04

**Authors:** Sandor Dudas, Renee Anderson, Antanas Staskevicus, Gordon Mitchell, James C. Cross, Stefanie Czub

**Affiliations:** a National and OIE Reference Laboratory for BSE, National Centre for Animal Diseases, Canadian Food Inspection Agency, Lethbridge, Canada; b Department of Veterinary Medicine, University of Calgary, Calgary, Canada; c National and OIE Reference Laboratory for Scrapie and CWD, Canadian Food Inspection Agency, Ottawa Laboratory Fallowfield, Ottawa, Canada

**Keywords:** Bovine spongiform encephalopathy, prion, bioassay, genotype, breed composition, in vitro conversion, pmca

## Abstract

Since the discovery of bovine spongiform encephalopathy (BSE), researchers have orally challenged cattle with infected brain material to study various aspects of disease pathogenesis. Unlike most other pathogens, oral BSE challenge does not always result in the expected clinical presentation and pathology. In a recent study, steers were challenged orally with BSE and all developed clinical signs and were sacrificed and tested. However, despite a similar incubation and clinical presentation, one of the steers did not have detectable PrP^Sc^ in its brain. Samples from this animal were analysed for genetic differences as well as for the presence of in vitro PrP^Sc^ seeding activity or infectivity to determine the BSE status of this animal and the potential reasons that it was different. Seeding activity was detected in the brainstem of the abnormal steer but it was approximately one million times less than that found in the normal BSE positive steers. Intra-cranial challenge of bovinized transgenic mice resulted in no transmission of disease. The abnormal steer had different genetic sequences in non-coding regions of the PRNP gene but detection of similar genotypes in Canadian BSE field cases, that showed the expected brain pathology, suggested these differences may not be the primary cause of the abnormal result. Breed composition analysis showed a higher Hereford content in the abnormal steer as well as in two Canadian atypical BSE field cases and several additional abnormal experimental animals. This study could point towards a possible impact of breed composition on BSE pathogenesis.

## Introduction

Bovine spongiform encephalopathy (BSE) is a prion disease of cattle. In the mid-1980s the United Kingdom had an outbreak of classical BSE which was linked to the recycling of meat and bone meal used in feed supplements for cattle [1]. The discovery of this oral transmission route raised concerns that humans consuming BSE contaminated beef might also be at risk of contracting the disease. These concerns were confirmed with the detection of the first cases of human BSE, now known as variant Creutzfeldt-Jakob disease (vCJD), which has been found primarily in young patients in the United Kingdom [2]. This report prompted countries around the world to increase BSE surveillance testing and to introduce numerous feed restrictions to prevent additional cases of BSE as well as increase food safety.

Surveillance testing for BSE is carried out using a number of internationally validated test methods. Most of these methods utilize the protease resistant properties of the BSE associated prion protein as a means to differentiate and detect infected animals. A slightly more sensitive method for routine testing uses a PrP^Sc^ specific ligand to capture BSE associated prion proteins. These methods have been extensively validated for the known types of BSE and function well for surveillance where samples are primarily from higher risk cattle [3]. Unfortunately, the emergence of new, biochemically distinct strains of BSE is always possible and the need for ultrasensitive methods has resulted in the development of techniques such as in vitro conversion and transgenic mouse bioassays [4].

For many years, a key method used to study transmissible prionopathies was bioassay. Initially, this was done in wild type rodent models resulting in extremely long incubation times. Advances in mouse transgenics resulted in the development of more sensitive and faster laboratory animal bioassay models. The transgenic bovinized mouse XV model (TgBov XV)(Freidrich Loeffler Institute, Germany) has been extremely useful in determining which host tissues contain BSE infectivity as well as elucidating the intricacies of BSE pathogenesis following oral challenge [5–7]. This mouse model not only expresses the bovine prion protein but it does so at a level several times higher than normal, significantly reducing the time to develop disease [8]. Similar transgenic models have been made for infection studies on chronic wasting disease and scrapie [9–11].

A second approach to detect low levels of misfolded prion proteins are in vitro conversion assays, namely real time quaking induced conversion (RT-QuIC) and protein misfolding cyclic amplification (PMCA). While the molecular level details are not fully understood, it is believed these methods rely on template directed misfolding whereby the presence of sample associated PrP^Sc^ acts as a seed to induce the conversion of native PrP^C^ provided as a substrate in the reaction. Successive rounds of incubation (believed to provide time for template directed misfolding of PrP^C^) and agitation/sonication (believed to cause disruption of aggregates creating additional seed templates) eventually result in the accumulation of detectable levels of misfolded prion proteins. Both of these methods have been successfully used to detect prion disease-associated amyloid seeding though PMCA has been more commonly used for classical BSE [12–14]. Due to the extremely high sensitivity of these methods, caution must be taken to ensure risks of contamination are minimized and replicate testing is often performed to increase confidence in the results. Testing routine surveillance samples using bioassay or in vitro conversion is not feasible but these methods are of great value for research. Methods such as these could be vital in detecting new TSE strains which might not have the same biochemical properties required for detection by current diagnostic test methods but still potentially pose a risk to animal health or food safety. The detection and characterization of humans and rodent models with prion diseases with varying amounts of protease resistant PrP^Sc^ [15–17], which may be missed by current diagnostic methods, support the need for continued development and use of non-traditional, highly sensitive detection methods.

The host-encoded prion protein is believed to be a primary component of the infectious agent; as such, host genetics greatly impact prion diseases in certain species including sheep, goats, deer and elk [9,11,18]. To date, no prion gene anomalies have been identified as having a major impact on classical BSE in cattle [19]. Knowledge of increased susceptibility and decreased incubation time, corresponding with prion protein levels, have led to the study of bovine prion gene (PRNP) regulatory regions [8]. In German cattle, a 23 base homozygous deletion allele in the 5ʹ flanking sequence of the prion gene is significantly over represented in BSE positive cattle. Functional assays have confirmed an increased PrP protein expression in this genotype [20,21]. European Holstein cattle were also shown to have more 23bp deletion homozygous animals in the BSE positive group but the significance was greatly increased when evaluating the animals based on several additional PRNP polymorphic sites [22]. When these same sites were explored in common German and Swiss breeds, the importance of the promoter/intron indels was confirmed in some breeds but was not significant in the brown breeds [23]. A breed effect was also seen in BSE cases from the north western German state of Lower Saxony. This study reported a 4 times greater likelihood that red Holstein cattle were found in the control group than in the BSE infected group [24]. The results seem to indicate that the PRNP gene regulatory region is significant when looking at BSE within a breed, but that other genes affect pathogenesis as demonstrated when multiple breeds are assessed.

The need to understand how different genotypes can affect BSE pathogenesis was highlighted in a recent oral BSE challenge experiment. Three steers were fed 100 g of strong positive BSE brain and 1 of the steer in this group developed the expected clinical signs but did not have detectable PrP^Sc^ or brain pathology. This steer had very low amyloid seeding capacity and no detectable infectivity based on in vitro conversion and transgenic mouse bioassay, respectively. This result has also been seen in oral BSE challenge cattle in larger scale studies performed at the Animal and Plant Health Agency, UK (APHA-UK). Genetic and breed associated reasons for this aberrant result are further explored and discussed, setting the stage for future work to help identify the specific cause of abnormal outcomes following BSE oral challenge in cattle.

## Results

The oral BSE challenged steers were assessed regularly for progressive clinical signs of neurological disease. Seven months prior to sacrifice, the first definitive clinical signs were noted in all 3 of the challenged steers. The animals were hyper-alert and reacted to touching in the head and neck area with repeated head tossing (Table 1). Three months later the steers started showing noticeable behavioural changes including anxiety and nervousness. At the last clinical exam all steers were very nervous, anxious and hypersensitive to touch, sound and light. Following this clinical evaluation additional handling was deemed a health risk to the animals and a safety risk to the animal care and veterinary staff so the animals were sacrificed and underwent an extensive post mortem examination and were sampled for testing.

**Table 1.** Clinical/neurological examination observations for the three oral challenge C type BSE steers. The clinical signs started at 45 months post challenge and progressed over the next six months. All three steers displayed similar and significant clinical signs of neurologic disease at 52 months post challenge and were sacrificed

Clinical/neurological examination observations			Animal ID
45 months post challenge		48 months post challenge	51 month post challenge
-Alert, moves ears frequently -Head tossing when touched on head -Head tossing in response to sound	**Steer 1 (29,014)**	-Nervous, defensive, kicks and pushes at side of squeeze -Very difficult to examine -Lame when leaving barn after examination	-Very alert, nervous, anxious -Head tossing, flinches suddenly, jerky reaction of the head -Flinches during light and sound test -Hyper-excited; neurological evaluation could not be completed -Sacrificed: 52 months post challenge
-Head tossing when touched on neck only	**Steer 2 (29,055)**	-Alert, nervous, salivating -Anxious when touched on head and neck -Vigorous kicking in response to touching back legs -Fear of camera, cautious of obstacles	-Excited, very nervous, salivating -Head tossing and jerking of the limbs -Extremely sensitive to tactile stimulus -Repeated hyper-reactivity to sound -Kick response when touched on trunk -Sacrificed: 52.5 months post challenge
-Alert, salivating -Head tossing when touched on head and neck -Up and down repeatedly in the squeeze	**Steer 3 (29,034)**	-Alert, nervous, salivating -Up and down in the squeeze -Difficult to examine	-Very alert, nervous, anxious, weary, salivating -Head tossing constantly, flinches suddenly, continuous movement -Sensitive to bright light -Hyper-excited and difficult to exam due to constant movement -Sacrificed: 52 months post challenge

**Figure 1.**
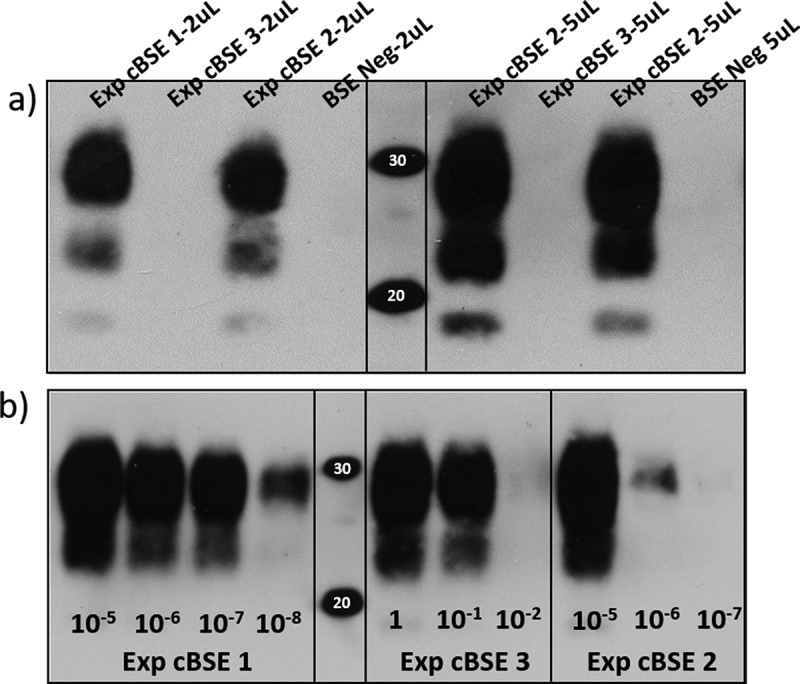
A) Western blot immunodetection of PK resistant prion proteins in the brain samples of the 3 oral challenge steers. Exp. cBSE 1 (29,014) and Exp. cBSE 2 (29,055) both have a strong positive signal with 3 immunoreactive bands at 30kDa and below. Exp. cBSE 3 (29,034) has no immunoreactivity in western blot. Western blot immunodetection of PK resistant PMCA products in different dilutions of brain homogenate used to spike into the PMCA reactions. b) Round 3 PMCA seeding activity/PK resistant PrP is detected in low dilutions for Exp. cBSE 1 (29,014) and 2 (29,055) indicating a high concentration of seeding units. Exp. cBSE 3 (29,034) has much less seeding activity

Brain tissues from the obex region were homogenized and tested using the Applied Biosystems Priostrip (ThermoFisher, Canada). Experimental steers 1 and 2 tested as high positive with optical densities greater than 7000, while the abnormal animal (steer 3) tested baseline negative with a value of 0 (test P/N cut-off OD = 112) (Table 2). To detect the presence of a PK sensitive PrP^Sc^, IDEXX Herd Check EIA was used to test samples from the three steers. The amyloid specific ligand-based test detected steers 1 and 2 as strongly positive but steer 3 gave a similar result to the known BSE negative control animal (Table 2). Various brain regions stained for histopathology and immunohistochemistry (IHC) were as expected for the two Priostrip and IDEXX positive steers. IHC detected prion protein aggregates throughout the brainstem of rapid test positive animals. Histological changes were also present including neuronal shrinkage/death and glial cell activation. The third steer had no notable BSE pathology and did not have immunohistochemical staining to indicate the presence of BSE PrP^Sc^ aggregates. PTA Western blot was evaluated for molecular weight, glycoform ratio and antibody reactivity which all showed the BSE prions in the 2 positive challenge steers were classical BSE (results not shown). Blot results for the third animal remained negative with no detection of protease resistant PrP in the brain of this animal, even after PTA purification (Table 2). Brain tissue homogenate from each of the steers was also tested for amyloid seeding capacity. Protein misfolding cyclic amplification (PMCA) detected seeding activity in all three of the challenged steers. The two previously tested positive steers seeded amyloid formation after one round of amplification and had detectable amyloid to dilutions of more than 10–6 after three rounds of amplification. Experimental steer 3 also seeded protease resistant amyloid formation but, importantly, not until the 3rd round of amplification and even then it was limited to the 10–1 dilution (Figure 1). Confidence in the PMCA results was reinforced by additional PMCA testing which also detected amyloid seeding in round 3 for steer 3 colliculus and cerebellar peduncles. Cervical spinal cord and thalamus from this animal remained negative in all three rounds of amplification. All BSE negative control homogenates remained negative in all three rounds of amplification.

**Table 2.** Molecular diagnostic test results for the three cBSE experimental steers as well as a negative and positive control animal. The positive/negative cut-off is listed for the Priostrip and IDEXX ELISA runs. PTA WB (phosphotungstic acid western blot) is visually interpreted, +++ is indicative of three very strong immunoreactive bands (one for each glycoform) that are difficult to separate due to the intensity of the signal

**Sample ID**	**Priostrip OD** **(p/n cut-off 60)**	**IDEXX ELISA OD** **(p/n cut-off 0.180)**	**PTA WB result/type**
Exp. cBSE 1 (29,014)	7141	3.24	+++/classical
Exp. cBSE 2 (29,055)	7388	3.44	+++/classical
Exp. cBSE 3 (29,034)	0	0.051	Negative/na
Exp. Negative (29,059)	0	0.056	Negative/na
CA #19 cBSE (872)	7816	3.57	+++/classical

**Figure 2.**
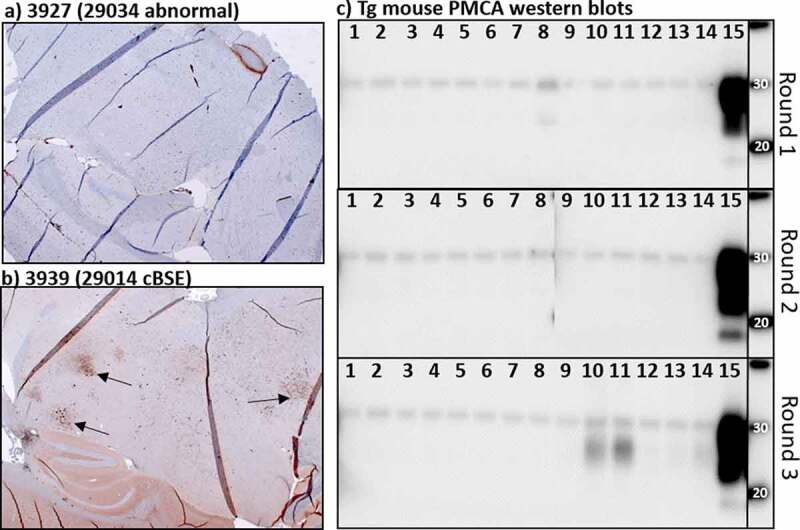
IHC and PMCA results from the Tg Bov XV mouse brains after being challenged intra-cranially with Exp. cBSE 1 (29,014) and Exp. cBSE 3 (29,034). a) Immunohistochemical staining of prion protein aggregates using anti-prion antibody F99. Some non-specific staining is seen around blood vessels but no aggregates are detected. b) Prion protein aggregates are detected in a number of areas in this transgenic mouse brain (arrows). c) Western blot detection of PK resistant prion proteins indicative of seeding activity in the brains of the mice tested. Mice challenged with Exp. cBSE 1 seeded PMCA formation of PK resistant PrP in all rounds (Sample 15). None of the Exp. cBSE 3 challenged mouse brains seeded PMCA conversion of PrP to PrPSc indicating no transmission of seeding activity from the index animal. Samples 1–12 3927 to 3938 (Exp. cBSE 3 (29,034) challenged mice), Sample 13: 3947 (29,059(BSE-), 595dpi), Sample 14: 3948 (29,059(-), 596dpi), Sample 15: 3940 (29,014(BSE+), 299dpi)

TgBov XV mice challenged with brain tissue from steer 1 developed clinical neurologic disease at 342 ± 13.5 dpi. Two mice from the negative control challenge group and one mouse from the group challenged with steer 3 brain homogenate were sacrificed due to inter-current disease at 338, 382 and 313 dpi, respectively. The remaining 15 mice from the control and steer 3 challenge groups survived for >410 dpi before they were sacrificed due to unrelated health issues or at the experimental endpoint (>550 dpi). All six mice challenged with steer 1 brain homogenate tested positive for protease resistant prion aggregates on western blot and with immunohistochemistry (Figure 2). All of the steer 3 and negative challenged mice tested negative for BSE using the PTA western blot. Non-specific reactivity was seen in some of the steer 3 challenged mice using immunohistochemistry but no BSE specific staining was identified. One of these BSE negative challenged mice had weak immuno-reactivity on PTA western blot but retesting this animal with a no primary antibody control blot revealed this reactivity was not prion protein but rather anti-mouse secondary antibody non-specific binding. Age-related pathology was seen in the brain tissues of the long incubation mice but there was no indication of significant neuronal death, gliosis or brain atrophy characteristic of prion disease [25].

Protein misfolding cyclic amplification was performed on all the mouse brains inoculated with experimental steer 3 homogenate to detect the presence of amyloid seeding in the absence of PrP^Sc^. Brain homogenate from mice challenged with tissue from experimental steer 1 seeded amyloid formation in all 3 rounds of PMCA (Figure 2). Negative control mouse seeded reactions did not produce protease resistant prion aggregates. The reactions seeded with brain from steer 3 brain challenged mice also failed to seed amyloid formation in any of the rounds of amplification. Round 3 samples from animals 10, 11 and, to a lesser degree, the negative control mouse (lane 14) do have a non-specific reactive product with a lower molecular weight than expected. This is sometimes seen in known BSE negative samples after multiple rounds of amplification and does not indicate the presence of amyloid seeding proteins in these samples.

**Figure 3.**
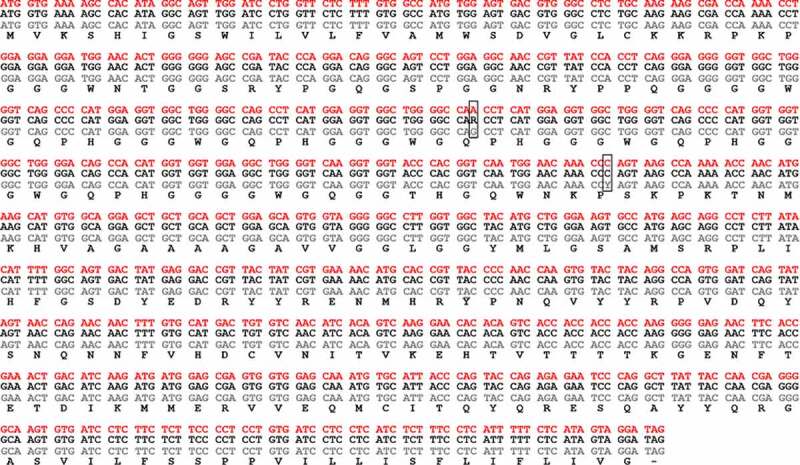
Nucleotide base sequence alignment and amino acid translation of the 3 oral cBSE challenged steers. Exp. cBSE 1 (29,014) grey text, Exp. cBSE 2 (29,055) black text, Exp. cBSE 3 (29,034) red text. Only 2 single nucleotide polymorphisms were identified in Exp. cBSE 1 but both are silent and do not result in an amino acid change

**Figure 4.**
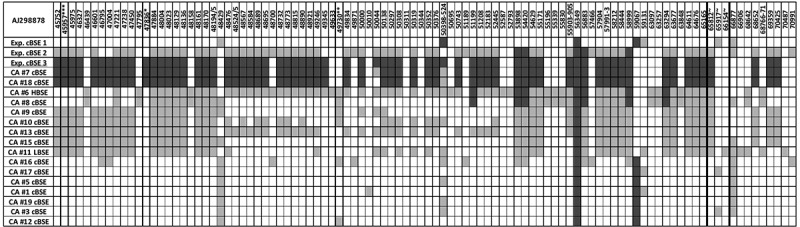
Variable SNPs in the coding and noncoding region of the PRNP gene of the 3 experimental BSE steers and 16 of the Canadian BSE field cases. SNP comparisons and numbering are based on PubMed reference sequence AJ298878. SNPs that are homozygous different than the reference sequence are shaded in dark grey, heterozygous SNPs are light grey and homozygous SNPs matching the reference sequence are unshaded. *23bp indel, **12bp indel, ***unstudied indel site, ~coding sequence SNPs

In order to understand the difference observed in disease outcome/pathology, we first assessed if the abnormal steer (steer 3, 29,034) had a different PRNP gene sequence. Amplification and direct Sanger sequencing of the prion gene open reading frame from each of the steers showed only two nucleotide differences, both in experimental steer 1 (29,014), with no change in the amino acid sequence in any of the animals (Figure 3). Next generation sequencing of the entire bovine prion gene revealed that experimental steer 3 did have a different non-coding nucleotide sequence compared to the other two steers. While experimental steer 1 primarily matched the sequence of DDBJ/EMBL/GenBank accession number AJ298878 and experimental steer 2 was a heterozygous match, the abnormal BSE steer was almost completely homozygous for the opposite genotype at most of the SNP loci (Figure 4). To better understand the potential significance, we examined the genome sequence of 16 Canadian field cases of classical BSE. The results showed that the PRNP genotype of steer 3 was extremely similar to two field cases (CA#7 and CA#18). BSE field case CA#18 was unremarkable while CA#7 was the youngest of all the Canadian BSE cases at 4.2 years of age. Both of these field cases demonstrated classical brain pathology so it seems unlikely that the non-coding polymorphism is the primary factor causing the abnormal outcome following BSE oral challenge in experimental steer 3.

To determine if non-PRNP gene polymorphisms contribute to altered BSE pathogenesis, we performed whole genome SNP genotyping. SNPs can be used to estimate the fraction of each breed present in mixed breed animals [26]; as an initial assessment, this data was used to determine the breed composition of the BSE-challenged steers and the Canadian BSE field cases. Despite the small sample size, this analysis demonstrated that atypical and abnormal BSE animals had a higher fraction of Hereford breed composition than normal BSE animals (Table 3). Experimental steer 3 (29,034), Canada’s two atypical BSE cases (CA#11 and CA#6) and two additional older classical BSE field cases (CA#9 and CA#8) all had a Hereford breed composition of 14.5% or more. In contrast, all other Canadian BSE field cases and normal BSE experimental cattle had a Hereford breed compositions of <9%.

**Table 3.** Fraction of the breed composition that is Hereford for the three experimental BSE steers and 16 of the Canadian BSE field cases and the BSE result for each of these animals. Composition Hereford is a fraction out of a total of 1.0

Identification	Composition Hereford	BSE result
CA#11 LBSE (19,216)	0.95227	atypical LBSE
CA#6 HBSE (811)	0.21051	atypical HBSE
CA#9 cBSE (2498)	0.18377	unique Can BSE FC
**Exp. cBSE 3 (29,034)**	**0.15502**	**abnormal cBSE**
CA#8 cBSE (814)	0.14526	longest inc. Can BSE FC
**Exp. cBSE 1 (29,014)**	**0.08575**	**normal oral cBSE**
**Exp. cBSE 2 (29,055)**	**0.03393**	**normal oral cBSE**
CA#17 cBSE (1386)	0.01677	normal Can BSE FC
CA#3 cBSE (45)	0.01494	normal Can BSE FC
CA#19 cBSE (872)	0.01457	normal Can BSE FC
CA#15 cBSE (13,364)	0.00809	normal Can BSE FC
CA#5 cBSE (809)	0.00646	normal Can BSE FC
CA#18 cBSE (1349)	0.00579	normal Can BSE FC
CA#12 cBSE (3222)	0.00385	normal Can BSE FC
CA#16 cBSE (4357)	0.00256	normal Can BSE FC
CA#13 cBSE (10,821)	0.00251	normal Can BSE FC
CA#1 cBSE (387)	0.00003	normal Can BSE FC
CA#7 cBSE (812)	0.00003	shortest inc. Can BSE FC
CA#10 cBSE (9655)	0.00003	normal Can BSE FC

## Discussion

Transmission of classical BSE has been linked to feeding BSE contaminated, animal origin feed supplements to young animals. When using this route for experimental BSE challenge studies, animals can have variable incubation times and sometimes even abnormal results. In a recent study at the Canadian Food Inspection Agency Lethbridge Laboratory, three steers challenged orally with 100 g of C type BSE positive brain material developed clinical symptoms of disease in a similar time frame but when sacrificed and tested for BSE, one of the steers did not have detectable PrP^Sc^. One would anticipate the large challenge dose and extended incubation period would result in PrP^Sc^ detection in the brain of all three steers. Detection of amyloid seeding using PMCA suggests that perhaps experimental steer 3 was infected but required additional time to accumulate a detectable amount of PrP^Sc^ in the central nervous system. Alternatively, perhaps another anomaly, such as host genetics, resulted in such a low level of PrP^Sc^ accumulation that it was undetectable on traditional diagnostic tests and only became detectable after multiple rounds of in vitro amplification. Unfortunately, we can only speculate whether the animal would have developed full blown disease since the answer could only have been determined by letting the animal continue to incubate past its sacrifice date.

Perhaps the most sensitive way to detect the presence of a transmissible spongiform encephalopathy in an animal or human prior to pathology and PrP^Sc^ accumulation is to use a bioassay sensitive for the prion disease of interest. Injecting TgBov XV mice with the brain material of experimental steer 3 did not cause clinical neurologic disease, PrP^Sc^ accumulation or neuropathology. Even PMCA amplification was unable to detect signs of infection in the steer 3 challenged mouse brains. This result indicates that if infectious BSE prions were present, they were not at a level infectious to the transgenic mice. Previous comparisons suggest that these mice are approximately 10 times more sensitive than cattle for the detection of BSE infectivity [27]. With this in mind it is safe to say, at the point of sacrifice at least, steer 3 contained negligible infectivity, if any at all. This result provides evidence that if other, similar abnormal BSE cases test negative in surveillance programs, they pose little or no risk to animal health.

These findings raise the question why the BSE outcome in steer 3 was so different from the other animals? One difference noted was the PRNP genotype. Experimental steer 3 had unique genetic sequence at a number of polymorphic sites in the prion non-coding region compared to the other 2 steers. Further comparing this unique genotype to 16 of the Canadian BSE field cases found that 2 classical BSE cases detected in Canada had similar genotypes. There was also no correlation with the experimental steer 3 genotype and long incubation field case animals, in fact one of the PRNP genetic matches had the shortest incubation out of all of the Canadian cases (CA#7, T06-812, 4.2 yrs old).

Breed composition analysis showed that steer 3 contained more Hereford breed composition compared to the others. Interestingly, analysis of the Canadian BSE field cases demonstrated animals with higher Hereford breed composition (>14%) present with atypical disease presentation and/or an extended incubation. This included the single Canadian H and L type atypical BSE cases (CA #6, 16.5 and CA#11, 13.7 yrs old, respectively), the oldest classical BSE field case (CA#8, T06-814, 8.6 yrs old) and a somewhat abnormal animal detected at 6.6 years (CA#9, T07-2498). This last animal is considered abnormal because it was the only Canadian BSE field case with moderate to low PrP^Sc^ deposition in the brainstem. This may indicate that this field case was not yet at full blown disease and required a longer incubation placing it in the extended incubation group. It is also possible that the Hereford breed has delayed PrP^Sc^ deposition or is more efficient at removing the aberrant protein from the brain leading to the results seen in CA#9 and steer 3. While this is somewhat speculative, the fact remains that this CA#9 is 18% Hereford and had significantly less brain-associated PrP^Sc^ compared to the other similar aged Canadian classical BSE field cases. A previous experimental result also lends support to the potential impact of breed composition on BSE. To amplify classical BSE from the first cases of BSE in Canada, brain homogenate was injected intra-cranially into 5 steer calves in 2005. Of these calves, 4 had 20% or more Hereford breed composition and one was <10% Hereford. This low Hereford animal was the first to reach full clinical disease and had high levels of PrP^Sc^ in its brain at sacrifice. The other animals took approximately 10% longer (2–3 months) to reach clinical disease and had similar levels of brain PrP^Sc^ at the time of sacrifice. This is a different inoculation route and a small sample size, but the results further support an impact of breed composition on classical BSE progression.

While high dose oral BSE challenges of cattle resulting in clinical animals with no detectable brain PrP^Sc^ are uncommon, it has been observed by others. The APHA-UK has been a leader in BSE challenge experiments and they have also seen a small number of BSE oral challenge animals exhibit clinical signs of BSE which, when sacrificed and tested, were negative for PrP^Sc^ (personal communication, Dr Timm Konold). Unfortunately, because these animals tested negative, samples were not retained. Had tissue been available, it would have been interesting to perform PMCA to determine the presence or absence of amyloid seeding. Availability of this tissue would have also allowed us to determine the breed composition of these abnormal animals to see if they were different when compared to normal BSE animals in these studies.

Alternative explanations for the abnormal results of the APHA-UK and our study are certainly plausible. It is possible that these PrP^Sc^ negative, BSE challenged cattle had altered gut conditions that resulted in the destruction or clearance of the BSE positive homogenate prior to uptake by gut associated lymphoid tissue resulting in no infection at all. This explanation does not, however, account for the fact that both steer 3 and the APHA-UK animals had clinical signs consistent with BSE in the expected incubation time. Prion disease research in humans and rodent models has found strains that cause clinical disease but have very low or undetectable levels of protease resistant PrP^Sc^ [15–17]. While it is possible that a similar phenomenon could occur in cattle, we would anticipate that such a bovine prionopathy would still result in detectable brain pathology and would likely contain infectivity sufficient to cause disease and/or pathology in the TgBov XV mice.

To our knowledge, this is the first study exploring potential reasons for unexpected results in cattle challenged orally with classical BSE. While initial testing suggested that steer 3 was negative for BSE, in vitro conversion demonstrated the presence of amyloid seeds that could be amplified from the brainstem, although at very low levels. Transgenic mouse bioassay did not, by contrast, detect infectivity in this brain tissue. Based on the evidence to date, it appears that differences in the PRNP gene itself do not fully account for the abnormal presentation but that other genetic differences are important. Previous studies have indicated that breed effects can overshadow PRNP polymorphisms and breed has been identified as a risk factor for BSE in a German cohorts [23,24]. Breed composition analysis indicates that, in our small cohort of experimental animals and Canadian BSE field cases, a high Hereford breed composition corresponds with abnormal or atypical BSE. Further exploration of high density SNP genotyping used for the breed composition analysis will hopefully identify particular genomic regions and associated genes which may be contributing to the Hereford breed associated BSE abnormalities.

## Methods

### Cattle challenge

Steers from the Canadian Food Inspection Agency Lethbridge Laboratory (CFIA-LL) specific pathogen-free herd were selected and the PRNP gene open reading frame was sequenced to ensure the challenge animals did not contain any non-synonymous polymorphisms. At 15 months of age, three animals (#1 29,014, #2 29,055, #3 29,034) were each fed 100 g of strong positive classical BSE central nervous system tissue from cattle reaching clinical endpoint following first passage of a Canadian BSE field case. All challenged animals were housed as per the Canadian biosafety guidelines for prion disease challenged animals and protocols were submitted to and approved by the CFIA-LL animal care committee. Monthly evaluations were carried out to detect clinical symptoms. All three animals displayed progressive clinical signs of similar severity in the same time frame. The animals were sacrificed at 52 months post challenge and brainstem, at the level of the obex, was tested immediately using the Applied Biosystems Priostrip BSE testing kit (ThermoFisher, USA). Additional testing was done using the IDEXX Herd Check EIA (IDEXX Laboratories, USA), western immuno-blot, immunohistochemistry, histopathology, in vitro conversion and transgenic mouse bioassay to confirm the BSE status of the steers.

### Genome sequencing and breed composition

Brain tissue for the three steers and most of the Canadian BSE field cases (n = 19) was used for DNA sequencing of the 30Kb PRNP gene region. Tissue (0.04–0.08 g) was digested in 270 µL of buffer ATL (Qiagen, USA) with 30 µL of proteinase K (Qiagen, USA) overnight at 56°C shaking at 300 rpms. The protease was heat inactivated at 70°C for 20 minutes prior to adding 100 µL of additional buffer ATL and 500 µL of chloroform. Each sample was mixed by vortexing, incubated at 4°C for 1 hour and spun at 16,000 x g for 10 minutes at 4°C. The top phase was moved to a new tube and mixed with 320 µL of cold ultra-pure ethanol and 50 µL of 3 M sodium acetate. Samples were spun at 16,000 x g for 10 minutes at 4°C and the supernatant was removed. Pellets were washed with 70% ethanol and allowed to dry before re-suspending in 50 µL of 10 mM TRIS-HCl, pH 8.5.

To sequence the PRNP gene, PCR primers were designed to amplify 7 x ~ 5 kb overlapping fragments. This regions stretched from 47,038,544 to 47,068,498 on bovine chromosome 13 (DDBJ/EMBL/GenBank accession number NC_037340.1). PCR products were gel purified using the QiaEXII kit (Qiagen, USA) and each individual animal’s set of 7 PCR products were quantified using a Qubit Fluorometer, equalized and then pooled for Nextera XT library preparation (Illumina, USA). Each pool of PCR products was tagmented with a unique combination of index sequences, amplified, checked for quality and normalized. The normalized library pools were used as input DNA for sequencing with the MiSeq Reagent Kit version 3, 150 cycles (Illumina, USA) on the Illumina MiSeq Instrument. Raw sequencing results were screened for quality and assembled using Geneious Prime Software (version 2019.1). Sequence alignments were done with SciEd Central Clone Manager (version 9).

Genomic DNA was also sent to Delta Genomics/Neogen for SNP genotyping using the GeneSeek Genomic Profiler Bovine 150k chip (Neogen, USA). These genotyping results were used to determine the breed composition of each animal.

### Transgenic bovinized mice XV (TgBov XV) challenge

TgBov XV mice were acquired from the Freidrich Loeffler Institute (Isle of Reims, Germany). Mice were challenged intra-cranially with 30 µL of 10% w/v bovine brain homogenate, from steer #1, steer #3 or a BSE free steer, prepared in sterile saline solution (0.9% w/v). Mice were examined several times each week to ensure they were in good condition and to identify the onset of clinical symptoms. At the appearance of progressive clinical signs, the experimental endpoint or for humane reasons, mice were sacrificed and 2/3 of each mouse brain was fixed in 10% neutral buffered formalin while the remaining 1/3 was frozen. Formalin fixed tissue was used for immunohistochemistry and histopathology while frozen tissue was used for molecular testing and in-vitro conversion assays.

### Applied biosystems priostrip BSE test kit (thermofisher, USA)

Brainstem tissue from the three experimental steers and 16 Canadian BSE field case cattle were homogenized to 10% w/v in 1 X homogenization buffer. After allowing foam to settle, 100 µL of tissue homogenates was mixed with 50 µL of digestion solution and incubated at 48°C for 60 minutes. Digestion stop was added to inactivate the protease and surviving proteins were denatured by incubating the samples for 15 minutes with 150 µL of assay buffer. A portion of the denatured sample (12 µL) was transferred to an assay plate and mixed with anti-prion antibody bound to coloured latex beads (80 µL). The beads and digested/denatured homogenate samples were wicked up an immuno-chromatographic strip to detect the presence of protease resistant PrP^Sc^. As the bead solution travels up the strip, it passes an immobilized line of anti-prion protein-specific antibody and a second immobilized line of goat anti-mouse antibody that will capture the mouse antibody conjugates blue latex bead. Two blue lines represent a BSE positive sample while a single blue line indicates the test has functioned properly but the sample does not contain detectable levels of protease resistant BSE PrP^Sc^. The density of the colour at both of the antibody lines was quantified using the PrioScan analysis software. This program assesses the colour and a value is assigned based on the average darkness of a defined area of the antibody line (Priostrip OD value).

### IDEXX Herd Check EIA (IDEXX Laboratories, USA)

Various central nervous system regions from the three experimental steers were trimmed and homogenized as per the manufacturer’s instructions (0.25 to 0.35 g of tissue per IDEXX bead homogenization tube). A sample preparation plate was prepared by adding 30 µL of sample diluent to each well except the first 4 control wells. One hundred and twenty microlitres of sample homogenate was mixed with sample diluent and 100 µL was transferred to the assay plate along with duplicate wells of the kit positive and negative controls. IDEXX prion disease detection kits do not use proteinase K resistance to differentiate between normal and BSE positive samples; instead, they use an amyloid specific ligand which has been bound to the assay plate to selectively bind PrP^Sc^. The assay plate was incubated, washed and captured protein was exposed to conditioning solution to unmask epitopes for detection. Bound PrP^Sc^ was detected with a horseradish peroxidase conjugated anti-prion antibody. After incubation, the plate was washed a final time and colorimetric substrate was added. Plates were incubated in the dark, the reaction was stopped, and the optical density of the plate was read using IDEXX XCheck software (version 3.3).

### Phosphotungstic acid (PTA) precipitation

Frozen brain tissue from the experimental steers and known control animals was homogenized to 10% w/v in PBS pH 7.2, 0.5% DOC, 0.5% Triton X-100 using a ceramic bead homogenization system. Homogenates were sonicated for two pulses of 30 seconds at power level 9 in a cup horn sonicator (Qsonica, USA). Samples were spun at 2500xg for 5 minutes to pellet out large cell debris. Two hundred microlitres of supernatant was transferred to new tubes and proteinase K was added to a final concentration of 50 µg/mL. Samples were incubated at 55°C for 60 minutes, shaking at 300rpm. The digestion was stopped by adding PMSF to a final concentration of 4 mM and boiling samples at 95°C for 5 minutes. An equal volume of 4% (w/v) sarkosyl was added (final concentration of 2% sarkosyl) followed by the addition of sodium phosphotungstic acid solution (pH to 7.2 with sodium hydroxide) to a final concentration of 2% (w/v). Samples were placed at 37°C for 1 hour shaking at 750 rpm. Following incubation, the samples were spun at 14,000xg for 15 minutes at room temperature. The supernatant was removed and the pellet was resuspended in 25 µL of 1 X SDS PAGE sample buffer and boiled for 5 minutes at 95°C.

### Western immuno-blot (WB)

Boiled samples were loaded onto a 10–12% SDS-PAGE gel and proteins were separated by electrophoresis at 150 V for 60 minutes. After transferring proteins to 0.45 µM PVDF (Sigma-Millipore, USA), the membrane was blocked with 0.1% iBlock (ThermoFisher, Canada) and then probed with the anti-prion monoclonal antibody 6H4 (ThermoFisher, Switzerland). The membrane was washed and incubated with horseradish peroxidase conjugated goat anti-mouse secondary antibody (Abcam, USA). Immuno-reactivity was visualized via chemiluminescence by adding SuperSignal West Femto substrate (ThermoFisher, Canada), images were taken using the BioRad Chemi-Doc imaging system and band analysis was done using the BioRad Image Lab software (version 6.0.1).

### PMCA

Frozen brain tissue from the experimental steers and known control animals was homogenized to 10% w/v in phosphate buffered saline, 150 mM NaCl, 5 mM EDTA, 1% Triton X-100 and complete protease inhibitor cocktail (Roche, Canada) using a ceramic bead homogenization system. Dilutions of the sample homogenates were made by adding 10 µL of the sample to 90 µL of 10% BSE negative transgenic bovinized mouse brain homogenized in the same buffer. Sample tubes were placed in a sonicator cup horn and incubated at 37°C for 72 hours with 20 second bursts of sonication every 30 minutes. At the end of the 72 hours, 10uL of the round 1 sonicated samples was transferred to a new set of tubes containing 90 µL of 10% transgenic bovinized mouse brain homogenate. The new tubes were placed at 37°C and sonicated for the same duration and pattern as in round 1. This procedure was repeated one more time for a total of 3 rounds of 72 hours of amplification.

To test for the seeding of protease resistant PrP, 20 µL of each PMCA reaction was mixed with SDS to a final concentration of 0.15% w/v and proteinase K to a final concentration of 55 µg/mL. Samples were vortexed and incubated at 55°C for 60 minutes. PK digestion was stopped by adding PMSF (4 mM final conc.) and an equal volume of 2X SDS PAGE sample buffer and then boiling at 95°C for 5 minutes. The denatured samples were tested by western immuno-blot using the same protocol as above.

### Immunohistochemistry and histopathology

Following complete fixation of tissues in neutral buffered formalin, mouse brain samples were trimmed and processed to infuse the tissues with paraffin. Processed tissues were embedded in paraffin blocks and sectioned at 3 µm and adhered to positively charged microscope slides. Residual paraffin was melted away and tissue sections were stained with haematoxylin and eosin (H and E) or used for immunohistochemical (IHC) detection of prion protein aggregates.

Formalin-fixed paraffin embedded tissue sections mounted on slides were deparaffinized and hydrated in decreasing concentrations of ethanol before being placed in Gill II Haematoxylin for 2 minutes and 30 seconds. After rinsing in running tap water, slides were immersed in saturated lithium carbonate solution, rinsed again and placed in 70% ethanol prior to eosin staining. Slides were immersed in eosin for 1 minute and 30 seconds and dehydrated in increasing concentrations of ethanol and finally 3 xylene rinses. Slides were then cover slipped prior to examination using light microscopy.

For immunohistochemistry, slide fixed tissue sections were deparaffinized in xylene and rehydrated in decreasing concentrations of ethanol until ending in Milli-Q water. Endogenous peroxidases were quenched with a 10 minute incubation in 3% v/v hydrogen peroxide solution. Slides were rinsed in Milli-Q water and place in formic acid for 5 minutes and again rinsed in Milli-Q water. The slide pH was stabilized using Tris buffer pH 7.6 ± 0.1 prior to autoclave assisted target retrieval. After cooling to room temperature the slides were transferred to TBST before applying manual staining cover plates and inserting the slides and cover plates into staining racks. All slides were rinsed with 2 mL of TBST before blocking with 100 µL of 5% v/v normal goat serum in TBST for 15 minutes. Slides were probed with 6C2 (Wageningen University and Research, Netherlands) or F99 (VMRD, USA) by adding 100 µL of TBST diluted primary antibody and incubating overnight at 4°C. Slides were rinsed with 2 × 2 mL TBST wash before adding 100 µL of horse radish peroxidase conjugated anti-mouse antibody polymer to each slide (Dako Envision + Anti-mouse, Dako Agilent, USA). After 30 minutes the slides were washed 2 × 2 mL with TBST and were removed from the staining rack and rinsed 2 times in Milli-Q water. Slides were placed in freshly prepared 5 µg/mL 3,3ʹ Diaminobenzidine tetrahydrochloride hydrate (DAB) solution in 100 mM imidazole buffer (pH 7.1 ± 0.1) and monitored every 30 seconds until desired staining intensity was obtained. Slides were thoroughly rinsed in Milli-Q water, counterstained with haematoxylin and dehydrated before cover slipping to protect the tissue/staining during light microscope examination.
